# Discovering SBIRT implementation and training priorities: The National SBIRT ATTC Needs Assessment

**DOI:** 10.1186/1940-0640-10-S2-P13

**Published:** 2015-09-24

**Authors:** Dawn Lindsay, Tracy McPherson, Piper Lincoln, Danielle Scott, Holly Hagle

**Affiliations:** 1 Institute for Research, Education, and Training in Addictions (IRETA), Pittsburgh, USA; 2 NORC at the University of Chicago, Chicago, USA

## Background

In the summer of 2013, the National Screening, Brief Intervention and Referral to Treatment Addiction Technology Transfer Center (National SBIRT ATTC) launched a national Needs Assessment focusing on the implementation of SBIRT services.

The objectives are to the purpose of the Needs Assessment was to assess the current use of SBIRT in various settings, to examine SBIRT implementation models, and to determine training and technical assistance needs.

## Material and methods

The target audience for the Needs Assessment was past and present SBIRT grantees, including SAMHSA, NIH, HRSA and other federal organizations. These individuals would be more likely to have the ideal circumstances (i.e., funding) to implement SBIRT, and having had substantial experience implementing SBIRT, would be most able to reflect on SBIRT needs for the future. The National SBIRT ATTC compiled the recruitment list from the database of present or past awarded grants with SBIRT as a keyword on grants.gov, filtered for relevance. The final recruitment list was 182 organizations.

The Needs Assessment instrument was developed by members of the National SBIRT ATTC staff and reviewed by several individuals from the Advisory Board. The survey was administered electronically using FluidSurveys.

## Results

The top three identified areas of need for both training and technical assistance were "Reimbursement and coding for SBIRT," "Sustainability of SBIRT," and "Reporting Joint Commission performance measures". The survey identified the typical respondent as in a University setting and currently funded either by SAMHSA or NIH to conduct training, clinical services, and/or implementation of SBIRT at a system level.

## Conclusions

The Needs Assessment yielded critical information that will be utilized in the development of future National SBIRT ATTC initiatives focused on SBIRT implementation at the systems level, and ultimately increasing the number of settings that are using SBIRT. Future assessments will target other specific populations within the SBIRT community.

